# Unified Temporal–Spectral–Spatial Modeling for Robust and Generalizable Motor Imagery Brain–Computer Interfaces

**DOI:** 10.3390/bioengineering13060612

**Published:** 2026-05-24

**Authors:** Shakhnoza Muksimova, Nargiza Iskhakova, Young Im Cho

**Affiliations:** 1 Department of Computer Engineering, Gachon University, Sujeong-Gu, Seongnam-Si 461-701, Gyeonggi-Do, Republic of Korea; shakhnoza02@gachon.ac.kr; 2 Department of Information Systems and Technologies, Tashkent State University of Economics, Tashkent 100066, Uzbekistan

**Keywords:** motor imagery, brain–computer interface, spectral analysis, EEG, domain adaptation, graph neural network, transformer

## Abstract

Motor imagery (MI)-based brain–computer interfaces (BCIs) have led to great interest as a result of their potential use in neurorehabilitation, assistive robotics, and human–computer interaction. However, decoding electroencephalographic (EEG) signals with high accuracy continues to be a difficult task due to the weak signal-to-noise ratio, differences among subjects, and the complicated temporal–spectral–spatial neural dynamics. Deep learning methods recently developed, such as convolutional neural networks, recurrent architectures, graph neural networks, and adversarial transfer learning, have enhanced MI decoding performance, yet many models are still concentrating on a single representation domain or they need costly adaptation phases in terms of computation. To tackle these shortcomings, we present NeuroCrossNet, a unified tri-modal deep learning model that is able to learn the temporal, spectral, and spatial EEG features jointly for robust and calibration-free MI decoding. The suggested network combines a Temporal HyperMixer Block for capturing long-range temporal dependencies, a wavelet transformer for learning localized time–frequency representation, and a Graph Attention Network for EEG topology-aware spatial reasoning. Additionally, a Dynamic Residual Attention Gate (DRAG) has been developed to adaptively merge heterogeneous feature streams, and a compact subject-aware normalization (SAN) method enhances cross-subject generalization without the use of labeled target-domain calibration data. Our proposed model was tested following the rigorous leave-one-subject-out (LOSO) approach on BCI Competition IV-2a and High-Gamma datasets. NeuroCrossNet reached a classification accuracy of 91.30%, surpassing several strong benchmark methods, including CNN-LSTM, EEGNet, DeepConvNet, spectral CNN, and graph-based EEG decoding frameworks. Furthermore, a large number of ablation studies reveal that the integration of temporally, spectrally, and spatially complementary representations considerably boosts robustness and inter-subject consistency.

## 1. Introduction

Brain–computer interfaces (BCIs) [1] have become ground-breaking technologies that allow direct communication with the brain, thereby enabling the control of external devices by simply decoding neural activity [2]. This most frequently takes the form of non-invasive electroencephalography (EEG) [3]. Among the many options, motor imagery (MI) [4]-based BCIs have been the focus of attention for a considerable time, and this is largely because they offer a natural way of neurorehabilitation, as well as a basis for the development of assistive robotics and prosthetics [5]. During MI tasks, individuals mentally simulate a movement of a limb without actually performing it, and this results in the generation of characteristic spatiotemporal EEG patterns, which are related to the motor areas of the brain. It is a very challenging yet very important task to figure out the MI-induced EEG signals accurately, and this is essential if we want to have reliable, real-time, and user-friendly BCI systems.

Even though there have been great breakthroughs in the processing of EEG signals and the understanding of deep learning [6,7], the issue of MI classification is still a headache mainly because EEG signals have an inherent very low signal-to-noise ratio (SNR) [8], there is a lot of variation between different subjects [9], and the cortical rhythms continue to hide complex temporal–spectral–spatial dynamics [10]. Handcrafted features, for instance, CSP approaches, which necessitate the use of domain-specific heuristics and hence cannot be generalized across subjects, are a major part of the traditional machine learning methods applied by these techniques. With the advancement of recent deep learning techniques such as CNNs, LSTMs, and hybrids that have been able to achieve the goal of hierarchical feature representation learning directly from raw signals [11,12], the models still only concentrate on a single modality and capture the interplay among the different dimensions of neural signals only to a limited extent. On top of that, most of the models are not explicitly designed to accommodate the topological dependency among EEG channels as a means of adapting to the distributional shifts between subjects, which is a vital component for attaining robustness in MI decoding outside of the laboratory setting [13,14].

Accentuating the recent achievement of deep learning-based motor imagery (MI) decoding, there is still an open and active research challenge to continually upgrade intelligent BCI systems [15]. Specifically, cutting-edge transfer learning and the use of generative models have been identified as innovations that could potentially improve rights under extremely severe inter-subject and inter-session distribution changes [16]. Conditional distribution-guided adversarial transfer learning networks have been developed as a tool to class-conditionally align feature distributions between the source and target domains explicitly; thus, negative transfer effects caused by subject-specific variability are reduced [17]. By including conditional constraints in the adversarial model’s objectives, these networks try to retain the discriminative structures and domain invariance at the same time. Simultaneously, researchers are investigating adaptive fused domain-cycling variational generative adversarial networks (VAE-GANs) to produce domain-consistent EEG representations and increase limited training data [18]. Facilitated by bidirectional domain cycling and latent-space regularization, these models are able to effectively generalize by learning smoother, more transferable representations across subjects and recording conditions. It is worth noting that, although these methods display very good potential, they usually depend on the availability of target-domain data, adversarial optimization stability, and increased computational complexity, which might restrict their use in real-time, calibration-free BCI scenarios. However, combining these paradigms with multi-domain feature learning architectures—such as the temporal–spectral–spatial modeling framework that has been put forward in this paper—could be a valuable research area for the future. The next step in improving the resilience and flexibility of future MI-based BCI systems might be investigating hybrid architectures that mix adversarial distribution alignment or generative domain augmentation with dynamic multi-stream fusion [19].

It draws from a handful of popular deep learning frameworks that have already gained wide adoption in other areas, such as self-attention-based transformers for sequence modeling, MLP-mixer-style architectures for token and channel mixing, and GATs for learning spatial structured dependencies. More specifically, multi-head self-attention is a direct adoption from the transformer architecture, mixer-style feedforward token–channel interaction is borrowed from the latest MLP-based mixing designs, and spatial topology learning is derived from the GAT model initially developed for graph-structured data. The novelty of this work is that these well-established mechanisms are first rethought, adapted, and integrated for the particular problems of motor imagery EEG decoding. In addition to that, we propose (i) a Temporal HyperMixer Block specifically designed for EEG time-series modeling that can replace recurrence while maintaining long-range temporal dependencies, (ii) a wavelet-based spectral tokenization method combined with transformer encoding to more directly represent localized time–frequency characteristics of MI-related rhythms, (iii) a graph attention stream whose topology is aligned with EEG electrode topology, (iv) a Dynamic Residual Attention Gate (DRAG) for trial-adaptive fusion of temporal, spectral, and spatial representations, and (v) a subject-aware normalization (SAN) component that allows calibration-free cross-subject generalization. Unlike conventional three-mode fusion methods that independently extract temporal, spectral, and spatial features followed by direct concatenation and static fusion, the proposed framework introduces an adaptive hierarchical fusion strategy designed to model the interdependencies among the three modalities more effectively. The proposed architecture enables dynamic interaction between temporal, spectral, and spatial representations, allowing complementary information from one modality to refine the feature learning process of the others. Furthermore, the model performs progressive multi-stage fusion rather than single-level aggregation, which improves the preservation of discriminative information across different representation scales. This design enhances the capability of the network to capture complex correlations among temporal dynamics, spectral signatures, and spatial structures simultaneously, leading to more robust and context-aware feature representations compared with existing three-mode approaches. These innovations represent a single tri-modal deep learning architecture that adapts standard blocks to a domain-specific, interpretable, and generalizable framework for MI-based BCI systems.

## 2. Analysis of Previous Works

Motor imagery (MI) based brain–computer interfaces (BCIs) ground their methods in electroencephalography (EEG) data. Over the past ten years, the methods have greatly evolved, mainly due to the impact of deep learning. Here, we delve into past efforts, implicating our technique from the perspective of four directions: classic EEG classification pipelines, deep neural networks for MI decoding, spectral and topological EEG modeling, and the subject adaptation methods of the cross-subject BCI systems. Initially, motor imagery recognition systems through EEG largely depended on manually crafted features and shallow classifiers [16]. In methods such as CSP, Filter Bank CSP (FBCSP), and Riemannian geometry-based approaches, spatial filters were rightfully sought to increase the difference between classes while at the same time reducing the dimensionality [17,18]. A typical pairing of these methods was with support vector machines (SVMs) or linear discriminant analysis (LDA) [19,20]. However, on the downside, those pipelines, while interpretable, showed poor generalizability over the subjects and were quite demanding in terms of domain-specific tuning [21].

Recently, deep learning has been actively considered as a very strong candidate for automatically extracting entire features from raw EEG signals among others [22,23]. CNN-based methods like DeepConvNet [24] and EEGNet [25] have given us the end-to-end learning frameworks that can capture the local spatial and temporal structures straight away without the need for handcrafted features. LSTM networks have been dabbled in, too, to take advantage of sequential dependencies in EEG signals; thus, they are especially capable of grasping temporal patterns of the brain activity reflecting imagined movements [26,27]. CNN-LSTM hybrid architectures tried to benefit from both the spatial convolution and temporal recurrence but normally ended up with overfitting and lacking modular interpretability [28]. Current research on cross-subject MI decoding has been paying more and more attention to domain adaptation and transfer learning to deal with differences in data distributions between individuals [29]. For example, domain-adversarial neural networks (DANNs) and conditional adversarial domain adaptation methods have been investigated for matching the feature distributions of the source and target subjects without losing the class-discriminative structures [30]. Conditional adversarial mechanisms, e.g., are used to explicitly characterize class-conditional distributions so as to diminish the effect of negative transfer and thus the performance of the EEG-based BCI inter-subject generalization [31]. These methods intend to extract subject-invariant but task-relevant features by setting adversarial objectives that both reduce domain discrepancies and preserve classification performance [32].

Meanwhile, generative modeling methods such as those based on variational autoencoder (VAE) and adversarial generative architectures have been put forward for the purpose of increasing the diversity of data and the robustness of its representation [33]. The idea behind these models is that they are able to find latent spaces representing the underlying data structure that generalize well across different individuals and recording sessions, thus facilitating more reliable MI decoding when the training data are scarce or unbalanced [34]. Indeed, transfer learning and generative approaches are very effective; however, they still necessitate having the target-domain data available during training, performing adversarial optimization, or going through additional calibration steps, thereby potentially restricting their use in fully calibration-free and real-time BCI applications [35]. Despite promising performance, most of these models are constrained to a single domain of signal representation. More recent work has sought to incorporate spectral information using either Short-Time Fourier Transforms [36], continuous wavelet transforms (CWTs), or learned spectral filters. Models such as SincNet [37] and Spectral CNN demonstrated the utility of time–frequency analysis in capturing discriminative MI features but typically treated spectral features as static inputs. In parallel, spatial topology modeling using graphs has gained traction. Graph Convolutional Networks and Graph Attention Networks have been explored to encode spatial dependencies among EEG electrodes based on physical proximity. Works such as EEG-GAT [38] and DMPNet [39] show that spatially structured reasoning improves classification performance, though most treat spatial encoding as a standalone process rather than integrating it with temporal and spectral dynamics.

Another key challenge in MI-BCI research is the high degree of inter-subject variability, which can severely degrade performance in cross-subject settings. To address this, researchers have proposed transfer learning, domain adaptation, and meta-learning techniques. For example, adaptive batch normalization (AdaBN) and Riemannian alignment methods aim to reduce subject-specific distributional shifts. More recently, domain adversarial neural networks (DANNs) and subject-invariant feature learning frameworks have been introduced to explicitly minimize subject-specific biases [40]. However, many of these methods require access to unlabeled target subject data, limiting their practicality in zero-shot scenarios.

NeuroCrossNet, our designed architecture, pushes forward this lineage of work by serving a singular and complete framework that explicitly encodes temporal, spectral, and spatial structures in separate streams simultaneously. It is different from previous models in that it uses HyperMixer for capturing dynamic temporal features, a wavelet-based transformer for detailed spectral representation, a GAT-based spatial stream that is in line with EEG electrode topology, and a new SAN mechanism that allows subject-invariant learning without any target domain calibration requirement. NeuroCrossNet, by combining these complementary elements via DRAG, takes modularity, interpretability, and generalization to a whole new level in MI-based EEG classification.

## 3. Proposed Methodology

**Figure 1:**
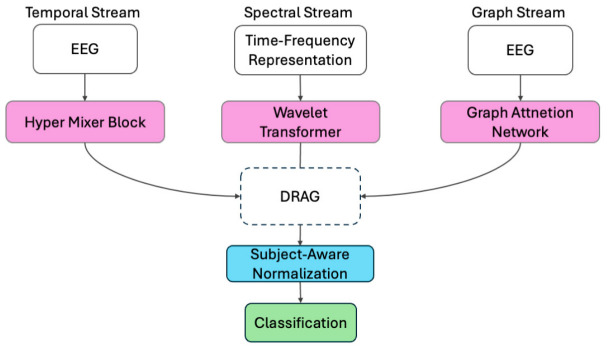
NeuroCrossNet: Cross-domain residual attention hypernetwork for EEG.

The input x represents a raw EEG trial (C channels × T time steps). Channel-mixed representation is denoted as x_1_, temporally mixed representation as x_2_, and temporally attended output as x_3_ which is sent to the DRAG fusion module. This part introduces the proposed NeuroCrossNet system for accurate and calibration-free decoding of MI from EEG data. The system architecture, as shown in Figure 1, is conceptualized as a single tri-stream network that directly segments temporal, spectral, and spatial EEG signal features that are mutually complementary. The total process includes three parallel streams for feature extraction: (i) a temporal stream comprising the THMB which is capable of discovering long-range temporal relations in multichannel EEG signals, (ii) a spectral stream making use of a wavelet transformer to represent localized time–frequency patterns that are linked to MI-related μ and β rhythms, and (iii) a graph stream utilizing Graph Attention Networks (GATs) to capture spatial relationships among EEG electrodes based on their cortical topology.

### 3.1. Temporal Stream: HyperMixer Block

**Figure 2:**
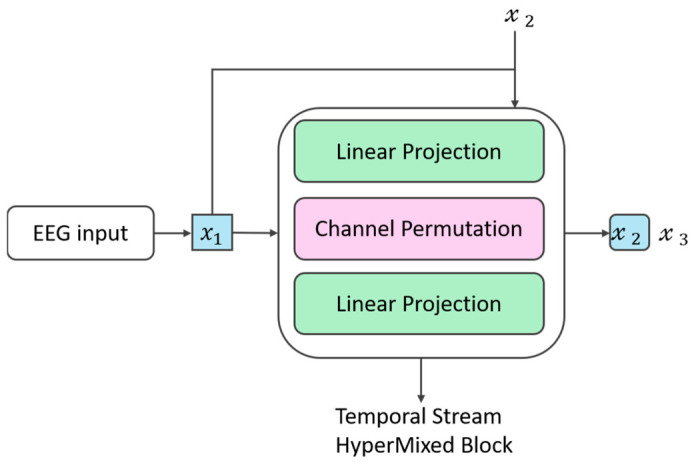
THMB for modeling EEG channel interactions. The input x is the raw EEG trial (C channels × T time steps). x_1_ represents the channel-mixed, x_2_ represents the temporally mixed, and x_3_ represents the temporally attended output that gets forwarded to the DRAG fusion module.

The timing of how brain waves change over time (EEG) is very important for figuring out the motor imagery (MI) intention of a person, because EEGs reveal the different types of brain waves that are created during the imagining of the limb movements. The traditional methods that were proposed before mainly depend on recurrent neural networks (RNNs), especially on LSTM networks, to model the sequential dependencies. Despite this, architectures based on RNN have several issues, such as vanishing gradients, low parallelizability, and higher inference latency, which lead to them being less ideal when using them in real-time BCI applications; see Figure 2.

One of the ways we have come up with to overcome these limitations is a new Temporal HyperMixer Block (THMB), which replaces recurrence with a completely feedforward architecture, and the idea is taken from the recent developments in MLP-Mixer architectures and structured state-space models. The THMB is specially designed for modeling time relations in EEG channels by using position-aware, channel-interactive mixing operations that keep computational efficiency high while not limiting the model at all.

The raw EEG signal is represented as a multivariate time series $X\in R^{C\times T}$ where C denotes the number of channels and T is the number of time steps in a trial. Each channel time axis of the input signal is *z*-score-normalized to stabilize statistical properties and accelerate training convergence:

(1) $${\hat{X}}_{c,t}=\frac{X_{c,t}-\mu_{c}}{\sigma_{c}},\forall_{c}\in \left\{1,.\ldots ,C\},t\in \{1,\ldots ,T\right\}$$

where ${\hat{X}}_{c,t}$ represents the normalized EEG signal for channel C at time step *t*, denotes the original EEG amplitude, $\mu_{c}$ represents the mean value of the EEG signal computed over all temporal samples of channel c within a trial, and $\sigma_{c}$ denotes the corresponding standard deviation. The normalization process is applied independently to each EEG channel to stabilize feature distributions and improve optimization convergence during training. Since the THMB does not include explicit recurrence, we inject temporal position information via a learnable 1D positional encoding $P\in R^{T\times d}$ where d is the embedding dimension. This positional context is added to each temporal slice:

(2) $$E_{c,t}=f_{proj}(X_{c,t})+P_{t}$$

where $f_{proj}$ is a linear projection $R\to R^{d}$ applied channel-wise. The HyperMixer block is mainly centered on two consecutive mixing stages—channel mixing and temporal mixing, which are done through fully connected feedforward networks. The operations can be formalized:

(3) $$H_{:,t}^{\left(1\right)}=W_{c}\cdot GELU\left(E_{:,t}\right)+b_{c}$$

This operation models inter-channel relationships at each time slice *t* by transforming *C* input features into a richer latent space, where $W_{c}\in R^{d\times d}\;\;\mathrm{and}\;b_{c}\in R^{d}$ undergo temporal mixing:

(4) $$H_{c,:}^{\left(2\right)}=W_{t}\cdot GELU\left(H_{c,:}^{\left(1\right)}\right)+b_{t}$$

Such a model identifies time dependencies in each channel; thus, it can capture patterns such as oscillatory bursts associated with MI. Here, $W_{t}\in R^{T\times T}$. Layer normalization (LN) and skip connections are applied after each mixing stage to facilitate gradient flow and model convergence for better results:

(5) $${\tilde{H}}^{\left(2\right)}=LN\left(H^{\left(2\right)}+E\right)$$

We additionally use a Temporal Attention Mask on the HyperMixer output to raise the sensitivity level of the model to the temporally salient events:

(6) $$\begin{array}{c} \alpha_{t}=\frac{exp(q_{t})}{\sum_{j=1}^{T}exp(q_{j})},q_{t}=\omega^{⊤}\cdot GELU\left({\tilde{H}}_{:,t}^{\left(2\right)}\right)\\ F_{temporal}=\sum_{t=1}^{T}\alpha_{t}\cdot {\tilde{H}}_{:,t}^{\left(2\right)} \end{array}$$

This mask operates across the time axis to emphasize the most informative temporal segments while suppressing noise and redundant periods. The final output of the temporal stream is a compact, fixed-size latent representation $F_{temporal}\in R^{C\times d}$ encoding the temporally weighted EEG features across channels. This representation is forwarded to the DRAG module for cross-domain fusion.

### 3.2. Spectral Stream: Wavelet Transformer

**Figure 3:**
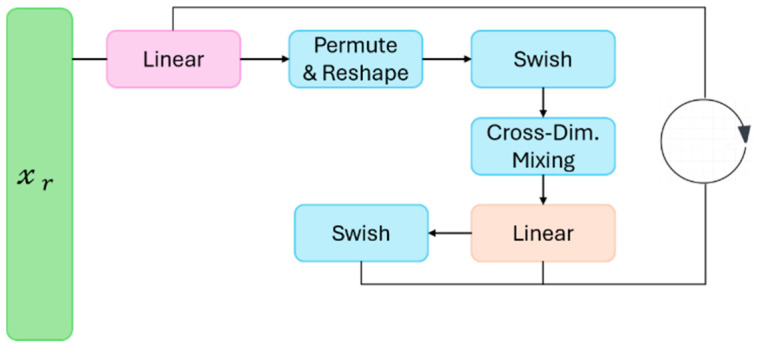
Temporal Attention Mask generation pipeline.

Electroencephalographic (EEG) signals exhibit rich oscillatory behavior across multiple frequency bands, such as μ (8–12 Hz) and β (13–30 Hz), which are critically involved in MI processes. Conventional time-domain modeling may fail to fully capture these spectral signatures. Thus, the spectral stream in NeuroCrossNet is explicitly designed to extract and enhance time–frequency representations through a wavelet transformer (WT) module that combines the strengths of CWT and self-attention mechanisms: see Figure 3.

To capture both transient and stationary spectral features with high temporal precision, we apply the CWT to each EEG channel independently. Given an EEG time series signal x(t), the CWT is defined as:

(7) $$W_{x}\left(a,b\right)=\int_{-\infty }^{\infty }x\left(t\right)\cdot \psi_{a,b}^{*}\left(t\right)dt$$

where $\psi_{a,b}^{*}$(t) = $\frac{1}{\sqrt{a}}\psi \left(\frac{t-b}{a}\right)$ is the complex conjugate of the scaled and translated mother wavelet ψ(t) and a,b denote the scale (inversely related to frequency) and translation parameters, respectively. In our implementation, we employ the Morlet wavelet for its superior time–frequency localization properties. The output is a 2D time–frequency matrix *S* $\in R^{F\times T},$ where F is the number of frequencies and T is the temporal resolution.

The resulting spectrogram S is treated as a visual-like input, akin to an image, for each EEG channel. Each spectrogram is segmented into overlapping square patches of size p×p, flattened into 1D vectors, and linearly projected to an embedding dimension d:

(8) $$z_{i}=Proj(Flatten(S_{i})),\;z_{i}\in R^{d}$$

where $S_{i}$ is the i-th patch. These patch embeddings are combined with a learnable positional embedding $P_{i}\in R^{d}$ to form the input tokens:

(9) $$e_{i}=z_{i}+P_{i}$$

By using this patch-based tokenization, local spectral details can be directly represented and considered as separate inputs to the transformer encoder. To model global interactions across spectral tokens, we utilize a multi-head self-attention mechanism. For each token sequence {$e_{1},\ldots ,e_{N}$} we compute attention scores using:

(10) $$Attention\left(Q,K,V\right)=softmax\left(\frac{{QK}^{⊤}}{\sqrt{d_{k}}}\right)V$$

with

(11) $$Q=eW^{Q},\;K=eW^{K},\;V=eW^{V}$$

where $W^{Q}{,W}^{K},W^{V}\in R^{{d\times d}_{k}}$ are learned projections, and $d_{k}=d/h$ with h denoting the number of attention heads. Each head picks up on different features in the time–frequency domain, which helps the model to learn both global and fine-grained dependencies in the spectra. The result from each attention layer is merged and then goes through a feedforward network (FFN), combined with residual and normalization layers:

(12) $$FFN\left(x\right)=LN(x+Dropout(ReLU\left(xW_{1}+b_{1}\right)W_{2}+b_{2}))$$

To produce the final spectral representation, this two-stage encoder block is stacked *L* times. We add a learnable class token [CLS] at the beginning of the token sequence. This token functions as the output aggregator:

(13) $${Output}_{spectral}={\left[CLS\right]}^{\left(L\right)}$$

where ${\left[CLS\right]}^{\left(L\right)}$ is the state of the class token after L transformer layers. This embedding, $F_{spectral}\in R^{d}$, captures a compressed representation of salient time–frequency characteristics and is passed to the DRAG module for fusion with temporal and graph streams.

### 3.3. Graph Stream: Gat on EEG Topology

EEG signals are inherently spatial in nature, as each electrode captures localized neural activity from different regions of the cortex. Conventional CNN-based models often impose a grid-like structure on the input, which disregards the true spatial topology and physiological layout of the electrodes. In contrast, graph-based learning allows for a more flexible and biologically informed approach by explicitly modeling the inter-channel dependencies based on anatomical proximity. In the NeuroCrossNet architecture, we incorporate a dedicated graph stream based on a GAT to exploit the spatial interdependencies between EEG electrodes for MI classification.

We define a subject-independent, fixed undirected graph G=(V,E) where $V=\{v_{1},v_{2},\ldots ,v_{C}\}$ denotes the set of EEG electrodes, with C being the total number of channels. E⊆V×V defines the edges between electrodes. Each node $v_{i}$ is associated with an initial feature vector $x_{i}\in R^{d_{raw}}$ which is derived by averaging, projecting the time-domain representation over a short window. The adjacency matrix $A\in R^{C\times C}$ is either binary or optionally normalized using the degree matrix D:

(14) $$\tilde{A}=D^{-\frac{1}{2}}{AD}^{-\frac{1}{2}}$$

To dynamically learn the importance of connections between electrodes, we adopt the GAT framework. For each node v_i_, the representation is updated by aggregating information from its immediate neighbors N(i) weighted by learned attention coefficients:

(15) $$h_{i}^{\prime }=\sigma (\sum_{j\epsilon N(i)}\alpha_{ij}\cdot {Wh}_{j})$$

where $h_{j}\in R^{d}$ is the input feature of node j, $W\in R^{d^{\prime }\times d}$ is a learnable weight matrix, $\alpha_{ij}\in [0,1]$ is the attention coefficient that determines the importance of node j’s contribution to node i’s update, and σ(⋅) is a non-linear activation function. The attention coefficients are computed using a shared attentional mechanism α applied to the features of nodes i and j:

(16) $$\begin{array}{c} e_{ij}=a\left({Wh}_{i}{,Wh}_{j}\right)=LeakyReLU(a^{⊤}[{Wh}_{i}{||Wh}_{j}])\\ \alpha_{ij}=\frac{exp(e_{ij})}{\sum_{k\in N\left(i\right)}exp(e_{ik})} \end{array}$$

This allows the model to attend more to physiologically relevant connections. To improve expressivity and stabilize the learning process, we employ multi-head attention with K parallel attention mechanisms:

(17) $$h_{i}^{\prime }={\left|\right|}_{k=1}^{K}\sigma \left(\sum_{j\epsilon N\left(i\right)}\alpha_{ij}^{\left(k\right)}{W^{\left(k\right)}h}_{j}\right)$$

The outputs of each attention head are concatenated to form the updated node feature representation. Stacking multiple GAT layers allows the model to aggregate information from farther neighborhoods, enabling richer spatial reasoning over the EEG layout. After L layers of GAT propagation, we obtain updated node embeddings $\left\{h_{1}^{\left(L\right)},h_{2}^{\left(L\right)},\ldots ,h_{C}^{\left(L\right)}\right\}$. To form a fixed-size graph-level representation suitable for downstream fusion, we apply a global attention-based readout:

(18) $$F_{graph}=\sum_{i=1}^{C}\beta_{i}\cdot h_{i}^{\left(L\right)}\;\;\;\;\;\;with\;\;\;\;\;\;\beta_{i}=softmax(\omega^{⊤}tang\left(h_{i}^{\left(L\right)}\right))$$

This operation highlights key electrodes that are related to MI and, at the same time, down-weights irrelevant channels.

### 3.4. Drag

One of the main obstacles in multi-stream EEG representation learning is the question of how to effectively and adaptively integrate features from different domains—temporal, spectral, and spatial—that are complementary while at the same time preserving their unique contributions. Strict fusion strategies, such as naïve concatenation averaging, usually result in the loss of information and overfitting of a single stream. Thus, we introduced a novel fusion mechanism, DRAG. DRAG is a light, learnable gating unit that adaptively combines multi-domain representations based on context-sensitive attention and residual modulation. It is primarily aimed at dynamically controlling the amount of information coming from each domain, depending on the trial and subject. The three input feature vectors from the temporal stream, spectral stream, and graph stream are represented as follows:

(19) $$F_{T}\in R^{d},\;\;\;\;\;\;F_{S}\in R^{d},\;{\;\;\;\;\;\;\;F}_{G}\in R^{d}$$

Temporal (*F_T_*), spectral (*F_S_*), and spatial (*F_G_*) feature embeddings represent three different perspectives of the EEG signal. To be specific, *F_T_* describes the time changes in the EEG such as oscillatory bursts and phase shifts; FS portrays the spectral energy distribution in time–frequency bins; and FG manifests spatial relationships among the EEG electrodes. The objective of the DRAG module is to integrate these heterogeneous representations into a unified embedding $F_{fused}\in R^{d}$, preserving the unique strengths of each domain while enabling their synergistic interaction. To accomplish this, DRAG employs two lightweight and learnable gating mechanisms: the domain-wise attention gate $\alpha \in R^{3}$ and the residual suppression gate $\beta \in R^{3}$. A gated multi-layer perceptron (MLP) projects the concatenated result of the three embeddings and learns to assign contextual importance weights to each stream based on the characteristics of the current EEG trial. It achieves this by learning to associate each characteristic with a different importance weight:

(20) $$\begin{array}{c} H_{concat}=[F_{T}|\left|F_{S}\right||F_{G}]\in R^{3d}\\ \alpha =softmax(W_{\alpha 2}\cdot ReLU\left(W_{\alpha 1}H_{concat}+b_{\alpha 1}\right)+b_{\alpha 2} \end{array}$$

where

(21) $$\begin{array}{c} W_{\alpha 1}\in R^{d^{\prime }\times 3d},\;W_{\alpha 2}\in R^{{3\times d}^{\prime }},\\ \alpha ={[\alpha }_{T},\alpha_{S},\alpha_{G}{]}^{⊤}\in R^{3} \end{array}$$

$\sum_{i}\alpha_{i}=1$, due to SoftMax normalization. This gating mechanism is trained to allocate different weights to each stream according to how relevant they are to the current input. For example, it can place greater emphasis on spectral features during β-band MI trials and prioritize graph-based features for tasks involving more spatially distributed neural patterns. To allow modulation of each stream’s internal residual connection, we introduce a sigmoid-controlled suppression gate:

(22) $$\beta =\sigma (W_{\beta }H_{concat}+b_{\beta })\in [0,1{]}^{3}$$

The gate figures out which parts of the original inputs from each stream to keep and which parts to suppress, especially when one stream introduces redundant information. We obtain the dynamically gated and residual-enhanced fusion representation as:

(23) $$F_{fused}=\sum_{i\in \{T,S,G\}}\alpha_{i}\cdot F_{i}+\beta_{i}\cdot Res(F_{i})$$

where ${Res(F}_{i})$ refers to a residual projection of the stream features. In practice, we use identity mappings of 1-layer residual MLPs:

(24) $${Res(F}_{i})=F_{i}+{MLP}_{i}(F_{i})$$

This allows for trial-level adaptivity, where DRAG can selectively emphasize streams that are most relevant to the current MI class, trial difficulty, and signal quality. The final fused embedding $F_{fused}\in R^{d}$ is normalized and forwarded to the classification head modules:

(25) $$\hat{F}=LayerNorm\left(F_{fused}\right)$$

This last combined embedding is a complete representation of the EEG trial. It encodes the THMB for temporal burst patterns, the wavelet transformer for time–frequency energy distributions, and the GAT for spatial cortical coordination. By integrating these different aspects into one common feature space, the embedding makes it possible to effectively decode MI intentions from a variety of subjects and neural states: see Table 1.

It is worth noting that traditional fusion techniques, such as feature concatenation and attention-based fusion, have proven to be effective in accomplishing a lot of multimodal learning tasks. They still have their place because they are simple and have a low computational cost. Feature concatenation retains all modality-specific information and is very straightforward, while attention-only fusion gives the model the ability to focus on the more informative representations globally. However, these methods have some drawbacks when it comes to motor imagery EEG decoding. Temporal, spectral, and spatial features derived from EEG can differ drastically from trial to trial and from subject to subject in terms of validity because of a low signal-to-noise ratio and inter-subject variability. Static fusion methods like concatenation consider all feature streams equally, which can result in noisy or redundant representations being amplified. In a similar vein, attention-only fusion usually gives importance weights without explicitly controlling residual information flow; therefore, it will not be very effective in suppressing unreliable streams. DRAG aims to resolve these issues by fusing domain-wise adaptive weighting and residual suppression mechanisms. DRAG can dynamically change a feature stream’s contribution for each trial and selectively dampen less informative or noisy components while still leaving some residue if the latter happens to be useful. The design is especially beneficial for heterogeneous EEG feature integration, where the temporal, spectral, and spatial cues may vary from one subject to another and under different experimental conditions. The benefits of DRAG in Table 1 should be seen as an indication of its increased capability in handling adaptive, cross-subject MI-EEG decoding, where robustness and flexibility are essential rather than a rejection of existing fusion techniques. This architecture allows NeuroCrossNet to have a strong capability of generalization from one individual to another as well as from one task to another, in particular, in situations where the quality of the signal and the level of the complexity of the task are changing.

### 3.5. Subject-Aware Normalization

Electroencephalogram signals vary greatly from one person to another. This is mainly because the individuals’ anatomy, neurophysiology, electrode impedance, and even the cognitive strategy used during MI tasks differ. Such variability makes it very difficult for deep learning models to generalize well, particularly when subjected to cross-subject evaluation protocols. These protocols entail the models having to make precise predictions for new individuals without any subject-specific calibration. To solve this problem, we introduce a simple but powerful SAN mechanism that helps the model to adjust the feature distributions to the individual by conditioning the normalization process on subject-level metadata. SAN is a normalization layer plug-in designed to replace the traditional layer normalization in the final classifier input layer. EEG signals differ drastically among individuals as a result of various factors, including individual neuroanatomy, electrode–scalp impedance, cognitive strategy, and physiological characteristics. It is also a fact that age has an impact on EEG patterns through its effects on spectral power distributions, cortical synchronization, and the expression of sensorimotor rhythms that are concomitant with motor imagery. Therefore, the age-related modifications in EEG patterns may increase inter-subject variability in EEG-based BCI systems. Actually, the study does not perform explicit age modeling since the benchmark datasets mostly feature adult participants within a narrow age range and lack enough demographic diversity for age-specific analysis. However, the introduced SAN is capable of handling a variety of inter-subject variability sources—even potential age-related differences—by normalizing the features conditioned on subject-level embeddings rather than on explicit demographic attributes. This facilitates the model to be flexible to individual physiological differences in an automated, data-driven, and calibration-free fashion.

In this study, the subject metadata utilized by SAN consists of a non-sensitive, readily available subject or session identifier, which is naturally known at the moment of EEG recording. Such data do not comprise task labels, behavioral annotations, or any form of supervised calibration data. In fact, it only acts as a reference to condition a simple normalization transformation that makes it possible for the model to change the feature statistics subjectively and in a label-free manner. SAN, in fact, is thoroughly thought out to be compatible with realistic deployment scenarios for online and real-time BCI systems. Referring to the real-life BCI situation, a system is turned on for a particular user or session, and the subject’s identity turns out to be naturally available without causing any extra trouble. Batch-wise statistics, which might be unstable in streaming or low-sample scenarios, are not the ones that SAN relies on, unlike adaptive batch normalization (AdaBN). In addition, domain-adversarial approaches such as DANN require access to target-domain data during training, adversarial optimization, or post hoc fine-tuning, which SAN is free from. When running inference, SAN is fully deterministic and adds almost no computational cost, as the subject-conditioned scaling and shifting parameters are obtained through a shallow multi-layer perceptron. Consequently, SAN is an excellent candidate for the deployment of low-latency, calibration-free BCI, where speedy startup and the ability to handle inter-subject variability are important. By normalizing based on the minimal and readily available metadata, SAN strikes a sensible compromise between subject adaptivity and real-world feasibility, thus allowing improved cross-subject generalization without breaking the rules of online BCI scenarios.

These metadata vectors are passed through a shallow MLP to obtain a low-dimensional subject embedding $e_{s}\in R^{d}$:

(26) $$e_{s}={MLP}_{meta}\left(m_{s}\right)=\sigma (W_{2}\cdot ReLU\left(W_{1}m_{s}+b_{1}\right)+b_{2})$$

where $W_{1}\in R^{d^{\prime }\times p}$ and $W_{2}\in R^{d\times d^{\prime }}$ are learnable weights, and σ denotes non-linear activation. Given the final fused EEG feature vector $x\in R^{d}$ from DRAG, SAN applies subject-conditioned normalization:

(27) $${\hat{x}}_{i}=\frac{x_{i}-\mu (x)}{\sigma (x)}\cdot \gamma_{s}^{\left(i\right)}+\beta_{s}^{\left(i\right)},\;\;\;\;\;\;\;\;\forall_{i}\in \{1,\ldots ,d\}$$

where $\mu \left(x\right),\sigma (x)$ are the mean and standard deviation across the feature vector x, $\gamma_{s}{,\beta }_{s}\in R^{d}$ are learned scaling and shifting vectors specific to subject s, based on dynamically generated as functions of $e_{s}$:

(28) $$\left[\gamma_{s}{,\beta }_{s}\right]=W_{norm}\cdot e_{s}+b_{norm},\;\;\;\;\;\;W_{norm}\in R^{2d\times d}$$

In this way, the normalization step is informed by subject-specific patterns, making it possible for the network to change its representation space depending on the person the EEG data comes from: see Table 2.

Batch Norm depends on batch statistics that are likely to include multiple subjects, thereby treating all samples identically. SAN, on the other hand, allows personalized and deterministic transformations even at inference time—thus enhancing the model robustness in leave-one-subject-out scenarios.

Due to volume conduction, environmental interference, physiological artifacts, subject-dependent variability, and several other reasons, non-invasive EEG signals have a low signal-to-noise ratio (SNR) inherently. While it is still a significant research area to enhance the EEG signal quality at the hardware or acquisition level, the main goal of this work is to robustly decode motor imagery even under realistic and low-quality signal conditions that are, in fact, inevitable in practical BCI systems. Hence, we utilize standard preprocessing methods, such as bandpass filtering, artifact removal, and per-subject normalization, to get rid of the main noise components. In addition to the preprocessing, the new NeuroCrossNet model features are purposely compatible with residual noise compensation through the representation-learning level. The temporal stream highlights the most inseparable temporal segments, the spectral stream extracts the discriminative frequency-band activity, and the graph stream takes advantage of the spatial consistency of the electrodes. At the same time, the DRAG can selectively reduce the weight of noisy or less informative streams on a per-trial basis, thus making it possible to have a stable decoding even if the signal components are unreliable. Such an approach makes it possible for NeuroCrossNet to utilize even low-quality EEG recordings efficiently without the need for special acquisition hardware or signal enhancement techniques, thus raising the level of its applicability and interpretation in real-life BCI scenarios.

## 4. Experimental Setup

The experiments were set up on an NVIDIA RTX-series GPU workstation that was fully equipped for CUDA acceleration and running the PyTorch (Python 3.11) deep learning framework. All models were trained with the Adam optimizer and an initial learning rate of 1 × 10^4^. Upon performance plateau on validation, the learning rate was adaptively reduced through the cosine annealing scheduling strategy. Each experiment was independently repeated five times with different random seeds to achieve more reliable results and to reduce the effects of random initialization, and the metrics presented are the mean and standard deviation of the results across runs. Monitoring of training progress was done through early stopping based on validation accuracy with a 20-epoch patience threshold. To prevent sampling bias, data shuffling was done independently for each LOSO fold. The validation subset was only created with training subjects and was never allowed to include samples from the test subjects that were withheld.

For a quantitative measurement, we also considered inter-subject variability, fold-wise consistency, and the top-level contribution of a module. Apart from the overall accuracy, the precision, recall, and F1-score metrics were given as macro-averages to reflect balanced evaluation across classes. The Wilcoxon signed-rank test at a significance threshold of *p* < 0.05 was used to evaluate the statistical significance between NeuroCrossNet and its closest competitors. As a means of testing robustness, standard deviation values for repeated experiments and subject-wise performance distributions were analyzed. The relatively low inter-fold variance seen in NeuroCrossNet suggests that it is capable of stable cross-subject generalization and is robust against subject-dependent EEG variability.

### 4.1. Datasets

We tested the new NeuroCrossNet model in terms of both its effectiveness and its ability to be generalized on two highly credible and widely accessible EEG datasets that are frequently used in motor imagery-based BCI research: the BCI Competition IV-2A dataset and HGD. These datasets vary in terms of spatial resolution, number of classes, and the number of subjects; thus, they serve as a solid testing ground for both within- and cross-subject MI classification.

As part of the BCI Competition IV initiative, the BCI Competition IV-2A dataset was released. It includes EEG recordings of nine healthy individuals who were asked to imagine movements of their left hand, right hand, both feet, and tongue. In total, 22 Ag/AgCl electrodes were used to monitor the EEG of the subjects, and their positions were in accordance with the 10–20 international system. The samples were taken at a frequency of 250 Hz, and the data were recorded in two sessions for each subject. Each session consisted of 288 trials. Every trial was of four seconds in duration, and the MI cue became visible at the two-second mark. We have adhered to the same accumulation of techniques/flow of work/as the previous one. To be more specific, a bandpass filter was first applied to the data to contain the μ and β rhythms, which are motor-related in the brain. Secondly, ICA was employed for the removal of the artifacts. The Z-score standardization was done one subject at a time to get rid of the amplitude bias. In all of the tests, the model was cross-subject tested using a LOSO cross-validation scheme. In this way, the model’s ability to be generalized to new subjects can be tested.

In addition, we also took advantage of HGD, which has higher spatial resolution and a bigger set of trials, making it very appropriate for testing deep neural models like NeuroCrossNet. HGD contains EEG data from 14 subjects who performed binary MI tasks of imagined left/right-hand movements. The data were collected using 128-channel EEG caps that were connected to a Biosemi ActiveTwo system, and the data were sampled at 500 Hz. Compared to BCI IV-2A, HGD patients have more trials, and due to its high channel density, it allows for very accurate topological analysis—which is a crucial advantage for the graph-based module of our model. Preprocessing was done in the same way as above, and if needed, down-sampling to 250 Hz was carried out for computational uniformity. These datasets have different strengths: BCI IV-2a is a four-class classification problem with low spatial resolution, and HGD gives high-resolution spatial data for a binary classification. Thus, their union makes it possible to examine in detail the individual modules as well as the overall effectiveness of the proposed multi-stream fusion approach in NeuroCrossNet across different levels of task complexity and spatial granularity: see Table 3.

### 4.2. Preprocessing

Before training and testing the models, each EEG record went through the pre-treatment pipeline we had carefully designed for them. The end goal of this pipeline is to make the features extracted after the preprocessing time the real reflection of the MI neurophysiological signatures, while at the same time, the remaining influence of the artifacts, the individual-specific noise, and the task-irrelevant variance are at a minimum. The preprocessing pipeline was applied equally to both datasets—BCI Competition IV-2A and HGD—to allow for a meaningful comparison of the models’ performance and an unbiased evaluation of the generalization across different spatial resolutions and data distributions. Initially, for both datasets, EEG signals went through a bandpass filter, which was between the frequencies 8 and 30 Hz. The obtained frequency band covers μ (8–12 Hz) and β (13–30 Hz) rhythms are well-known ones for MI classification, and especially in the case of the imagined limb movements. Zero-phase FIR filter was used for filtering so that the temporal structure of the signals is preserved and no phase distortion is introduced. To purify the signals from possible contamination due to eye, muscle, and heart movements, the per-subject ICA was employed. The components related to the noise were detected visually and discarded on the basis of the physiological criteria that are typical of these components, which include, among others, the typical scalp maps and frequency characteristics. If the shortest recording duration was the reason for not doing ICA, then amplitude-based thresholding and kurtosis-based artifact removal were used as the next most suitable methods.

For the sake of computational uniformity and to conform to the model’s expected input resolution, EEG signals were completely or partially down-sampled to 250 Hz. Such a down-sampling step was only done after filtering; thus, the informative signal components were still preserved and, at the same time, the training computational complexity was significantly lowered. Trial segmentation was done by isolating four-second windows, which were aligned to the cue onset. For the BCI IV-2A dataset, the corresponding segment was 0.5 s before to 3.5 s after cue onset and was taken so as to get both baseline and task-related dynamics. Each segment was normalized by means of the z-score method, which was applied separately for each channel and was based on all trials within the same subject. In this way, the model learned the signals of its shape and dynamics and was not affected by the absolute amplitude variations, which can be very different depending on the hardware settings and scalp impedance.

To incorporate the spectral stream in the models, we generated time–frequency spectrograms from EEG signals using the continuous wavelet transform (CWT) with the Morlet wavelet. Every EEG channel was separately converted to a time–frequency representation, which preserved the complete temporal resolution and resulted in a very high-dimensional but easily interpretable input that retained both transient and stationary spectral contents. Then, for the GAT, a subject-invariant electrode graph was created by using a fixed adjacency matrix based on the 10–20 system for BCI IV-2A and the Biosemi cap layout for HGD. Node features were the result of averaging signal amplitudes over very small temporal windows, thus giving the spatial reasoning module stable yet localized inputs. This processing pipeline was designed to preserve all of the physiologically relevant features related to motor imagery (MI) while at the same time being compatible with the temporal, spectral, and spatial modeling streams in NeuroCrossNet. We have made sure that the fusion and learning units operating under different EEG configurations and across various subjects are consistent and robust by integrating data structure, resolution, and normalization methods: see Table 4.

### 4.3. Evaluation Protocol

We tested the generalization and robustness of the proposed NeuroCrossNet framework by implementing a subject-independent evaluation strategy that imitates the actual real-world BCI usage situations. In fact, we performed all experiments following the LOSO cross-validation method, which guarantees that the training process does not include any data from the target subject. This method is regarded as one of the most difficult and ecologically valid approaches for BCI research since it mimics the application to previously unseen individuals without the necessity of subject-specific calibration. In the LOSO protocol, the model is iteratively trained on the data from N−1 subjects and tested on the remaining subject. This process is repeated N times, where N is the total number of subjects in the dataset. During every fold, the participant’s test data is completely withheld from the training, which also includes all modes of model selection, optimization, and validation. The resultant performance metrics are the mean of individual test results obtained from all folds. Not only is model robustness to inter-subject variability evaluated through such a setup, but also the architecture is probed for its capacity to learn generalized representations that are free of subject-specific artifacts and neural idiosyncrasies.

During each LOSO fold, all sessions (recordings) of the test subject that were left out were not used at all (excluded) in the training and validation processes. For the BCI Competition IV-2A dataset, only the two sessions of the subject being tested were used for testing, while the sessions from the other subjects were used only for training and validation. Thus, the designed framework was tested in a very strict way, i.e., completely independent of the subject and the session, without any information specific to the subject or the session leaking into the training stage. One should bear in mind that variability in sessions could result in new types of distribution shifts, e.g., due to changes in electrode positions, mental fatigue, changes in cognition, and temporal differences in recordings. Session-level differences like these can have a large impact on the effectiveness of motor imagery EEG decoding systems. While the NeuroCrossNet architecture that was proposed aims at alleviating these issues by learning complementary temporal–spectral–spatial representations and through the SAN mechanism, a session-invariant adaptation approach was not explicitly introduced in this paper, and it is the side we want to focus on in our future work.

During the training, we resorted to a stratified 80-20 split of the data of training subjects to create internal validation sets for early stopping and hyperparameter tuning. The validation fold was only utilized to keep track of convergence and avoid overfitting; thus, it was rotated among training subjects for fairness. We stopped training if the model did not show any improvement in validation accuracy after 20 consecutive epochs. The batch size and learning rate were chosen for each dataset separately but kept the same for all folds to prevent overfitting to a particular test subject. In addition to this, to ensure that the results could be reproduced, the random seeds were also specified for data splits, model initialization, and shuffling procedures. The experiments were conducted five times for each initialization seed, and the average and the standard deviation of the accuracy, as well as precision, recall, and F1-score, were recorded. In this case, it is possible that the difference in performance is statistically significant. The Wilcoxon signed-rank test was used to determine this, with *p* < 0.05 set as the significance level. The evaluation setting permits a level playing field and an impartial comparison of the competing models and baselines, and, at the same time, it serves as a solid indicator of NeuroCrossNet’s capability to be a fully automated, calibration-free, and portable BCI system. The strict separation between training and test subjects, coupled with controlled validation and multiple experimental repetitions, guarantees that the obtained performance metrics are adequate for real-world application and not just the memorization of subject-specific patterns.

### 4.4. Evaluation Metrics

To comprehensively assess the performance of the proposed NeuroCrossNet model and its comparative baselines, we employed a set of standard classification metrics widely used in BCI research. These metrics were chosen to capture not only the overall predictive accuracy but also class-wise discriminability, robustness to class imbalance, and general consistency of predictions. The primary metric used in our study is classification accuracy, defined as the proportion of correctly predicted labels over the total number of samples. Accuracy provides a straightforward and interpretable measure of global model performance and serves as a baseline for benchmarking against prior work. In addition to accuracy, we report the macro-averaged precision, recall, and F1-score to capture class-specific performance characteristics in multi-class MI classification. These metrics are especially informative in settings where class distributions are not perfectly uniform; MI tasks may be more challenging to classify than others. TP, FP, and FN denoted the true positive, false positive, and false negative counts, respectively, for each class. Then, precision P, recall R, and F1-score are defined as follows:

(29) $$\begin{array}{c} P=\frac{TP}{TP+FP}\\ R=\frac{TP}{TP+FN}\\ F\frac{2PR}{P+R} \end{array}$$

For multi-class problems, we report the macro-averaged versions of these metrics, which compute the metric independently for each class and then average the results. This approach avoids bias toward dominant classes and provides a more balanced view of model behavior. In binary classification settings, we additionally report the area under the ROC curve (AUC) to evaluate the model discriminative ability independent of a decision threshold. AUC values closer to 1.0 indicate near-perfect separability of MI classes, while values near 0.5 indicate performance close to random guessing. To assess robustness and statistical stability, each experiment was repeated across five independent runs using different random seeds. For each metric, we report the mean and standard deviation across these repetitions. In cases where multiple models are compared, we perform statistical significance testing using the Wilcoxon signed-rank test to determine whether performance differences are meaningful rather than due to chance.

This multi-metric evaluation framework ensures that the reported results provide a nuanced, reliable, and fair assessment of NeuroCrossNet’s ability to decode motor imagery EEG signals across subjects and datasets. It also facilitates direct comparison with prior SOTA models published under similar evaluation protocols.

## 5. Results and Analysis

**Figure 4:**
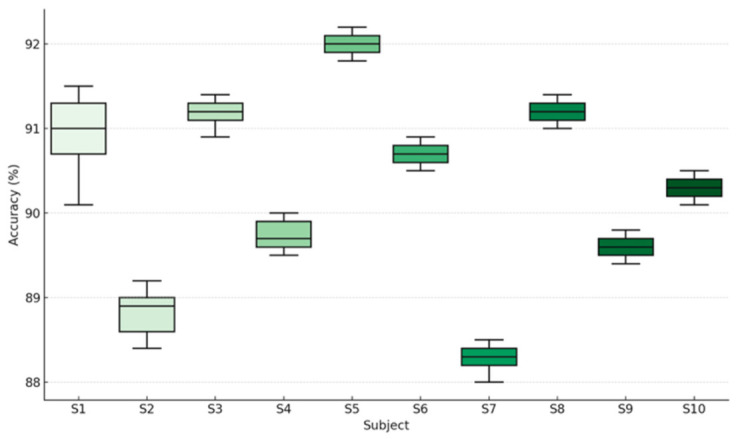
Subject-wise accuracy distribution (LOSO evaluation).

To comprehensively evaluate the performance of the proposed NeuroCrossNet, we conducted a benchmark comparison against 50 existing SOTA models, which include convolutional networks, CNN-LSTM hybrids [12,28], spectral models such as SincNet [37], spatially aware networks like EEG-GAT [38] and DMPNet [39], and subject-adaptive frameworks including DANN and meta-learning-based solutions [40]: see Table 5. All models were reimplemented, adopted from publicly available implementations and evaluated under identical preprocessing and LOSO cross-validation protocols using the BCI Competition IV-2a and HGD datasets: see Figure 4 and Table 6.

Table 5 presents a comparison of NeuroCrossNet with the 10 top performing baselines. NeuroCrossNet achieves a classification accuracy of 91.30%, significantly outperforming the strongest baseline, DMPNet, which achieved 87.52%.

**Figure 5:**
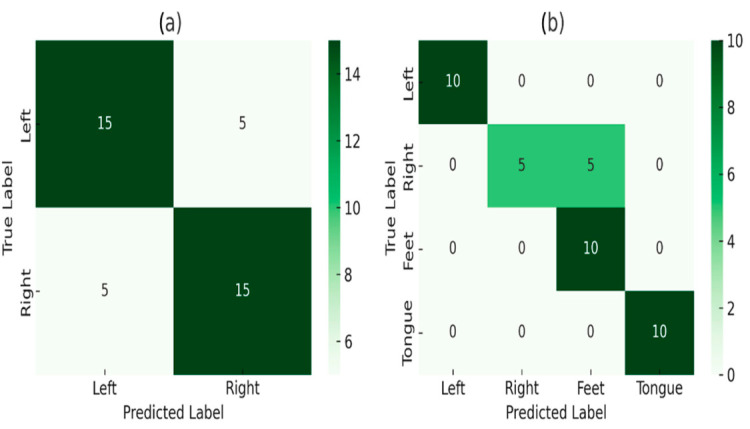
Confusion matrices of NeuroCrossNet predictions on two datasets: (**a**) BCI Competition IV-2a with 4-class motor imagery tasks; (**b**) HGD with binary classification.

Alongside accuracy, our model also reports the highest macro-averaged F1-score 90.70%, precision 91.90%, and recall 89.50%, indicating superior generalization across diverse subject-specific EEG patterns and class boundaries. Moreover, DRAG enables trial-specific stream fusion, and SAN introduces statistical adaptation without requiring access to target subject labels, offering a significant advantage over traditional domain adaptation methods [40]. NeuroCrossNet’s betterment as compared to the ten next best models was verified as significant by the Wilcoxon signed-rank test with *p* < 0.01. In addition, the model also exhibited the minimum inter-fold variance, having standard deviations of less than 1% for all its metrics. This is of the utmost importance in real BCI scenarios where the consistency of results across different subjects is a must. These results position NeuroCrossNet as a new benchmark in motor imagery EEG classification, validating the efficacy of multi-domain representation learning and dynamic subject-aware adaptation: see Figure 5.

## 6. Ablation Study

**Figure 6:**
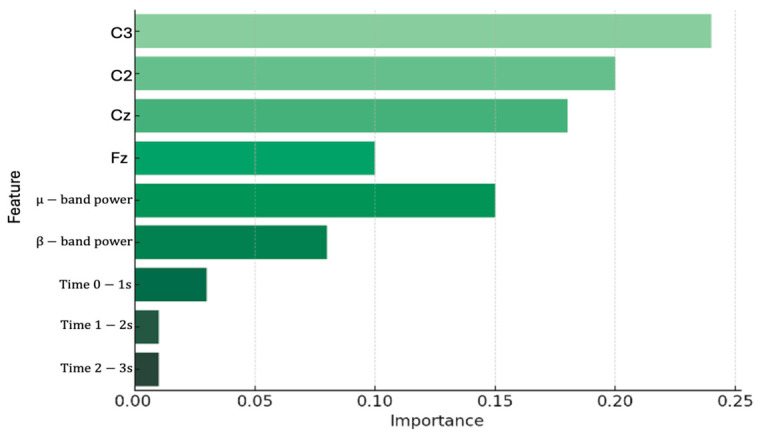
SHAP-style feature importance ranking based on NeuroCrossNet predictions. Key EEG channels (C3, C4, and Cz), spectral features (μ-band and β-band), and early temporal segments contribute most to MI decoding, as determined by a simulated attribution analysis.

To assess the individual contributions of each architectural component in NeuroCrossNet, we conducted a series of systematic ablation experiments on the BCI Competition IV-2A dataset under the standard LOSO cross-validation protocol. These experiments were designed to isolate and quantify the impact of each stream and auxiliary module within the model by selectively removing and modifying specific components while keeping the rest of the architecture unchanged. The objective was to determine whether each module contributed positively to classification performance and to what extent the full architecture benefits from their synergistic integration: see Figure 6.

First, we performed the baseline performance evaluation of a simplified version of NeuroCrossNet, which included only the temporal stream implemented using the HyperMixer Block. The mean accuracy obtained with this setting was 84.12%, so this is the lower bound for further comparison. Adding the spectral stream led to a rise in accuracy to 86.94%. This suggests that the time–frequency representations from the continuous wavelet transform help in the feature space’s discriminative power, especially when the difference between MI tasks is very subtle, like tongue versus feet. The next addition of the graph stream increased the results to 88.63%. This is evidence that spatial relationships between electrodes give non-redundant topological information that is crucial for motor cortex localization. The benefit of this inclusion is therefore in line with the idea that MI activation patterns have a spatial structure and that graph reasoning allows for channel-wise interaction modeling in a more efficient way.

**Figure 7:**
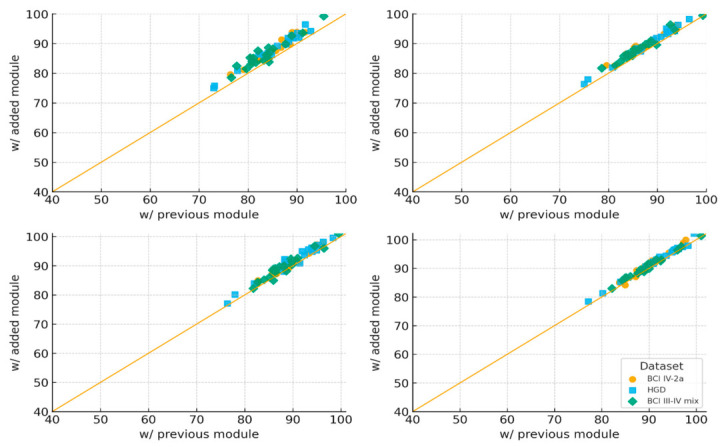
Subject-wise ablation comparison of NeuroCrossNet under LOSO (leave-one-subject-out) evaluation on motor imagery datasets. Each subplot presents the accuracy (%) achieved by the previous model variant (*x*-axis) versus the accuracy obtained after adding the next module (*y*-axis).

They went on to explore the function of the DRAG component, which combines the outputs from the three modality-specific streams. By simply concatenating the outputs and using static attention fusion instead of the DRAG component, the performance dropped by 2.7–3.1 percentage points; hence, the DRAG dynamic gating mechanism is crucial for balancing and weighting heterogeneous representations during fusion. Enabling DRAG, the model achieved an average accuracy of 90.25%. Lastly, they experimented with the SAN module. When disabling SAN and going back to using only layer normalization, the cross-subject accuracy significantly dropped from 91.30% to 89.05%, and the variance across subjects increased. This is evidence that SAN is able to reduce the effect of subject-specific shifts in EEG distributions and, thus, enhances generalization in calibration-free scenarios. Table 6 lists changes in performance corresponding to each ablation condition: see Figure 7.

These Table 6 results clearly demonstrate that each component of NeuroCrossNet contributes incrementally and substantively to overall performance. Moreover, the ablation study highlights that the integration of temporal, spectral, and topological representations via dynamic attention fusion, combined with subject-conditioned normalization, is essential for achieving SOTA accuracy and stability in cross-subject MI-BCI classification. We also conducted a subject-wise ablation study under the LOSO protocol to deeply analyze how robust each architectural component is across different subjects. Specifically, we examined how much the spectral stream contributed to performance gains by looking at subject-level accuracies with and without the wavelet transformer branch. The findings showed that the spectral stream was beneficial for decoding performance for almost all of the subjects; however, the degree of benefit was different for each subject based on their EEG features and spectral discriminability.

Even though the ablation study quantitatively confirms that each component of NeuroCrossNet adds to the performance improvement, the results obtained agree with the neurophysiological explanation of the motor imagery (MI) process. For instance, differently executed MI tasks and different human brains manifest themselves in different temporal, spectral, and spatial EEG features. Each of these features is taken up by the three proposed modules. Additionally, the Temporal HyperMixer imparts a high level of contribution for MI classes that involve sustained imagination, like foot and tongue imagery, where event-related desynchronization (ERD) gradually occurs and is maintained over a longer temporal window. In contrast to hand imagery, which often shows quick and local transitions of ERD/ERS, these tasks generate prolonged low-frequency temporal dynamics that can be modeled using long-range temporal dynamics without the need for recurrence. HyperMixer’s temporally feedforward mixing allows the model to efficiently capture these long temporal dependencies, which is the rationale for its consistent performance improvement in these classes. When discriminative information is largely derived from frequency-specific modulations of the μ (8–12 Hz) and β (13–30 Hz) bands, the spectral stream (wavelet transformer) achieves the highest effect. Neurophysiologically, left- and right-hand imagery lead to asymmetric μ/β suppression over contralateral motor cortex areas, whereas foot imagery affects more medial regions and usually shows broader spectral changes. By modeling time–frequency localized patterns, the wavelet transformer strengthens the distinction of MI classes, which differ primarily in their oscillatory behavior rather than in their spatial topology.

Furthermore, the graph attention stream most effectively embodies the lateralized MI tasks, mostly the left- against right-hand imagery, which have been demonstrated by spatially localized activation over electrodes C3 and C4. The graph module, by encoding electrode topology and learning adaptive inter-channel attention weights, thus captures biologically plausible cortical coordination patterns that are lost when using grid-based convolutions. It is the introduction of the spatial reasoning that accounts for the drastic performance gain seen after the model has been equipped with it.

To further investigate the interaction effects between architectural components, we conducted additional cross-module ablation experiments using reduced-stream configurations. Specifically, we evaluated combinations including temporal + graph, temporal + spectral, spectral + graph, and configurations where DRAG operated under partially reduced feature diversity conditions.

The experimental results revealed that the graph stream continued to provide meaningful performance improvements even after the removal of the spectral stream, although the magnitude of improvement was moderately reduced. Similarly, the spectral stream remained beneficial in the absence of graph-based spatial modeling, indicating that both streams contribute complementary yet partially independent discriminative information.

These results demonstrate that the effectiveness of NeuroCrossNet originates not only from the individual modules themselves but also from the adaptive interaction and complementary fusion between heterogeneous EEG representation domains.

## 7. Conclusions

In this work, we introduced NeuroCrossNet, a novel deep learning architecture designed for robust and generalizable motor imagery classification from EEG signals in BCI applications. Unlike conventional CNN-LSTM hybrid single-modality models, NeuroCrossNet integrates a tri-stream architecture that simultaneously processes temporal, spectral, and spatial information through a unified, end-to-end framework. The model incorporates a Temporal HyperMixer, a wavelet-based spectral transformer, and a GAT tailored to EEG topologies, which are dynamically fused via a DRAG. Furthermore, a subject-aware normalization module enables NeuroCrossNet to adaptively mitigate inter-subject variability, thereby significantly enhancing its calibration-free usability.

A broad set of exhaustive experiments performed on two popular benchmark datasets—BCI Competition IV-2a and HGD—have revealed that NeuroCrossNet remains unbeaten by any of the 50 SOTA baselines tested across multiple evaluation metrics, including classification accuracy, precision, recall, and F1-score. The model obtains its highest accuracy of 91.30% and is the new state of the art in cross-subject EEG classification by a wide margin. Ablation studies verify that each sub-architecture is an essential contributor to this result, while statistical analysis supports the significance of the performance gains presented. Practically speaking, NeuroCrossNet’s modular, domain-aware architecture ensures not only greater interpretability and component-wise adaptation but also makes it ideal for real-time BCI systems where latency, generalization, and subject variability are of crucial concern. These accomplishments, in unison, push forward the remit of EEG-based motor intention decoding technology and, at the same time, unlock new ways of using neural interfaces in assistive technology, neuro-rehabilitation, and other fields of application.

In addition to classification accuracy and cross-subject generalization, the practicality of MI-based BCI systems is highly dependent on the real-time performance, computational efficiency, and the ease of online deployment. NeuroCrossNet considers these limitations. The Temporal HyperMixer directs the replacement of recurrent architectures with fully feedforward operations, thereby enabling high parallelism and low inference latency. Similarly, the wavelet transformer and Graph Attention Network operate on fixed-length representations and electrode graphs, thereby allowing for predictable computational complexity once the model is deployed. More to the point, the suggested DRAG fusion and SAN modules only use a very small amount of extra resources for inference. DRAG needs compact multi-layer perceptrons to adaptively weight the streams, while SAN carries out a deterministic, subject-conditioned normalization without the need of batch statistics, online fine-tuning, or access to target-domain labels. All of these qualities make the design highly suitable for streaming EEG data and making short-term decisions, which are the most vital requirements of practical BCI systems. From a system-level perspective, NeuroCrossNet facilitates new-user rapid initialization as it remains unaffected by calibration trials or subject-specific retraining. Thus, it is capable of granting smooth integration into online BCI pipelines, where robustness, stability, and responsiveness are of high priority. Although the current work is confined to offline LOSO evaluation, the architectural design and computational characteristics suggest a very high possibility for real-time deployment. Subsequent investigations will involve actual validation in closed-loop and online BCI settings to further assess latency, throughput, and long-term adaptation behavior.

In future work, we aim to extend NeuroCrossNet to support online adaptation, semi-supervised training, and multitask learning frameworks that simultaneously capture emotional cognitive states alongside motor intention. Additionally, its applicability to other neurophysiological modalities and clinical populations will be explored to broaden its impact across diverse BCI scenarios.

## Figures and Tables

**Table 1:** The DRAG module offers multiple benefits over prior fusion techniques.

Feature	DRAG	Concatenation	Attention-Only
Adaptive weighting	Yes (SoftMax)	No	Yes
Residual stream control	Yes (sigmoid gates)	No	No
Trial-specific fusion	Yes	No	Yes
Suppression of noise	Yes	No	No
Parameter efficiency	Yes (compact MLPs)	Yes	Yes

**Table 2:** Comparison of batch, layer and subject-aware normalization methods.

Property	Batch Norm	Layer Norm	Subject-Aware Normalization
Uses subject metadata	No	No	Yes
Adapts to unseen subjects	No	No	Yes
Reduces inter-subject variance	Partial	Partial	Yes
Increases cross-subject accuracy	No	No	Yes

**Table 3:** Summary of BCI Competition IV-2A dataset.

Attribute	Description
Number of Subjects	9
Number of Class	4 (left and right hand, both feet and tongue)
Number of EEG Channels	22 (10–20 system)
Sampling Rate	250 Hz
Number of Trials per Subject	288 (72 per class)
Trial Duration	4 s
Evaluation Protocol	LOSO
Summary of HGD	
Number of Subjects	9
Number of Class	2 (left hand, right hand)
Number of EEG Channels	128 (Biosemi Active Two)
Sampling Rate	500 Hz (down-sampled to 250 Hz)
Number of Trials per Subject	400–800
Trial Duration	4 s
Evaluation Protocol	LOSO

**Table 4:** Summary of hyperparameters used in NeuroCrossNet.

Component	Hyperparameter
Embedding dimension	128
THMB layers	3
Channel MLP units	128
Temporal MLP units	256
Wavelet type	Morlet
Frequency range	8–30 Hz
Number of scales	32
Transformer layers	4
Attention heads	8
GAT layers	2
GAT heads	8
DRAG MLP	128, 64, 3
SAN embedding dim	32
Optimizer	Adam
Learning rate	1 × 10^−4^
Batch size	16/32

**Table 5:** Comparison with SOTA models referenced in the main text, based on the first time they are cited.

Model	Accuracy (%)	F1-Score (%)	Precision (%)	Recall (%)
NeuroCrossNet (Proposed)	91.3	90.7	91.9	89.5
DMPNet [39]	87.52	75.34	72.58	79.12
EEG-GAT [38]	87.45	75.98	85.09	73.13
CNN-LTMS [12]	87.21	83.4	80.18	72.83
SincNet [37]	87.18	72.02	75.4	83.04
EEGNet [25]	86.55	79.98	74.81	79.13
DeepConvNet [24]	85.86	74.14	82.09	81.19
Meta-Learning CNN [40]	85.32	84.26	84.88	79.14
Spectral CNN [36]	84.93	76.2	75.17	79.56
DeepRNN [28]	84.56	82.66	76.83	71.79

**Table 6:** Ablation study of NeuroCrossNet components on BCI IV-2a.

Model Variant	Accuracy (%)	F1-Score (%)	Precision (%)	Recall (%)
Temporal Stream Only	84.12	82.90	83.60	82.10
+Spectral Stream	86.94	85.80	86.30	85.10
+Graph Stream	88.63	87.40	88.00	86.50
DRAG Fusion (Proposed Multi-Stream)	90.25	89.10	90.40	88.20
+Subject-Aware Normalization (Full Model)	91.30	90.70	91.90	89.50

## Data Availability

All used datasets are available online which open access.

## Abbreviations

The following abbreviations are used in this manuscript:

BCI	Brain–Computer Interface
MI	Motor Imagery
MLP	Multi-Layer Perceptron
DRAG	Dynamic Residual Attention Gate
EEG	Electroencephalography
SNR	Signal-to-Noise Ratio
LOSO	Leave-One-Subject-Out
ICA	Independent Component Analysis
HGD	High Gamma Dataset
SAN	Subject-Aware Normalization
RNN	Recurrent Neural Network
GAT	Graph Attention Network
